# Maternal investment in relation to sex ratio and offspring number in a small mammal – a case for Trivers and Willard theory?

**DOI:** 10.1111/j.1365-2656.2009.01574.x

**Published:** 2009-09

**Authors:** Esa Koskela, Tapio Mappes, Tuuli Niskanen, Joanna Rutkowska

**Affiliations:** 1 Department of Biological and Environmental Science, University of JyväskyläP.O. Box 35, Jyväskylä FI-40014, Finland; 2 Centre of Excellence in Evolutionary Research, University of JyväskyläP.O. Box 35, Jyväskylä FI-40014, Finland; 3 Institute of Environmental Sciences, Jagiellonian UniversityGronostajowa 7, 30-387 Kraków, Poland

**Keywords:** cost of reproduction, litter size manipulation, nest defence, polygynous mating system, sexual size dimorphism

## Abstract

1. Optimal parental sex allocation depends on the balance between the costs of investing into sons vs. daughters and the benefits calculated as fitness returns. The outcome of this equation varies with the life history of the species, as well as the state of the individual and the quality of the environment.
2. We studied maternal allocation and subsequent fecundity costs of bank voles, *Myodes glareolus*, by manipulating both the postnatal sex ratio (all-male/all-female litters) and the quality of rearing environment (through manipulation of litter size by −2/+2 pups) of their offspring in a laboratory setting.
3. We found that mothers clearly biased their allocation to female rather than male offspring regardless of their own body condition. Male pups had a significantly lower growth rate than female pups, so that at weaning, males from enlarged litters were the smallest. Mothers produced more milk for female litters and also defended them more intensively than male offspring.
4. The results agree with the predictions based on the bank vole life history: there will be selection for greater investment in daughters rather than sons, as a larger size seems to be more influencial for female reproductive success in this species. Our finding could be a general rule in highly polygynous, but weakly dimorphic small mammals where females are territorial.
5. The results disagree with the narrow sense Trivers & Willard hypothesis, which states that in polygynous mammals that show higher variation in male than in female reproductive success, high-quality mothers are expected to invest more in sons than in daughters.

## Introduction

The issue of sex-biased parental investment has become one of the most examined areas in evolutionary biology and a variety of life-history patterns has been recognized under which differential allocation to sons and daughters could be adaptive (Charnov 1982). The majority of research is inspired by the Trivers & Willard (1973) theory, which proposes that in conditions where one sex gains more from extra parental resources than the other, the parents with relatively more resources will bias their allocation towards the sex with greater fitness returns. When applied to species in which males have a higher variation in reproductive success than females (like in polygynous mammals), the theory gives exact predictions that mothers in good condition should invest more in sons (so-called narrow sense T–W hypothesis; Cockburn, Legge & Double 2002). Trivers & Willard’s original idea have been developed over the years to broader statements (Charnov 1982; Trivers 1985; Frank 1990), and it is important to note that nowadays, the T–W hypothesis can be considered as one explanation in a long list of (not mutually exclusive) adaptive models of sex allocation (11 hypotheses listed in Cockburn *et al.* 2002).

Sex biases in maternal investment may be manifested in birth sex ratio (sex ratio allocation) as well as in maternal expenditure (energy allocation) during pre- and postnatal life (Hewison & Gaillard 1999; Cameron & Linklater 2002). Thus far, the topic of sex allocation in mammals has been addressed mainly in primates and in ungulates (e.g. Kruuk *et al.* 1999; Brown 2001; Sheldon & West 2004; Holand *et al.* 2006). There have been surprisingly a few studies conducted on small mammals, most of which in laboratory rodents (review in McGuire & Bemis 2007) and only a few studies have used wild small mammals (reviews in Clutton-Brock & Iason 1986; Hardy 1997; Bond, Wolff & Krackow 2003; Koskela *et al.* 2004; review in McGuire & Bemis 2007). Examining energy allocation between male and female offspring during nursing would be an interesting aspect of study because size at independence is often found to be important for the future performance of individuals (Roff 1992).

Due to the polygynous nature of mammalian mating systems (Clutton-Brock 1989), male–male competition for access to females has been proposed to drive the evolution of male body size, and thus the male-biased sexual size dimorphism (SSD) that prevails in most mammals (Weckerly 1998). However, it may be surprising to notice that a lack of SSD or even reversed SSD is common in microtine rodents (Ralls 1976). There is a scarcity of studies that seek to identify the selective pressures causing these patterns (reviewed in Schulte-Hostedde 2007). Bondrup-Nielsen & Ims (1990) analysed SSD for 21 populations of microtine rodents (11 species) and found that the female-to-male home-range size ratio explained most of the variation in SSD. They proposed that the sex competing for a resource, whether it is the females competing for space (territory) or males competing for groups of females, will be under selection for larger size (but see Ostfeld & Heske 1993). In the yellow-pine chipmunk (*Tamias amoenus*, J. A. Allen 1890), sex differences in the relationship between body size and fitness were consistent with female-biased SSD evident in this species (Schulte-Hostedde, Millar & Gibbs 2002). Clearly, more empirical work is needed to determine the possible sex biases in maternal investment in species over different circumstances, and further identify the selective forces underlying the observed sex-allocation patterns.

Here we aim to experimentally examine optimal maternal energy allocation between male and female offspring using the bank vole (*Myodes glareolus* Schreber 1780). As described below, the study system enables us to test whether maternal investment in this polygynous mammal is biased towards the sex that has larger variation in reproductive success (males), even though the opposite sex is predicted to benefit more from increased investment (larger body size) in terms of reproductive success and survival. The bank vole mating system is truly polygynandrous, showing higher variation in male than in female reproductive success (Mills *et al.* 2007a). However, directional selection does not seem to exist for greater body mass in males. This is demonstrated by the weak (or absence of) SSD (see Bondrup-Nielsen & Ims 1990 and Material and methods) and a stronger selection for male bank vole plasma testosterone level compared to body size (Mills *et al.* 2007a, b, 2009). Moreover, in the enclosure study by Klemme *et al.* (2006), male bank voles of different body masses did not differ in their reproductive success. In contrast, a large body size has been found to be beneficial for the reproductive success and survival of female bank voles repeatedly. The body size at birth positively affected the probability of breeding and the size of the first litter in the field (enclosures), and also corresponded with an earlier age at maturation in the laboratory (Mappes & Koskela 2004). Moreover, body weight at weaning positively predicted the probability of breeding already during the summer of their birth (Mappes, Ylönen & Viitala 1995b). Finally, a larger weight at weaning explained the better survival probabilities over winter among females but not males (Koskela 1998). Taken together, these studies suggest that, in the bank vole, a large body size could be more beneficial for female rather than male fitness; therefore it would then be more profitable to invest in daughters than sons.

These hypotheses were addressed using an experimental design involving simultaneous manipulation of the postnatal litter sex ratio (all-male and all-female litters) and the rearing environment (by changing the original litter size by −2 or +2 pups) of bank vole females by cross-fostering newborn offspring. Relative differences in maternal investment in sons and daughters were measured with four parameters: (i) milk production (which is the most energetically demanding process of maternal care; Lee 1987); (ii) growth of the pups (to estimate how maternal investment translates into offspring quality); (iii) mother’s defensive behaviour (to assess the relative value of the offspring; Montgomerie & Weatherhead 1988); and (iv) the mother’s subsequent reproductive success (to estimate the potential fitness costs it encounters).

## Materials and methods

### Study species

The bank vole, which is a common rodent in the Palaearctic region, experiences multi-annual fluctuations in its abundance throughout most of Finland (Kallio *et al.* 2009). During the breeding season in central Finland (May–September), females give birth to up to four litters with four to eight pups per litter (Koivula *et al.* 2003). A characteristic of *Myodes* species, including the bank vole, is that they are rather monomorphic as adults or even show reversed SSD (females larger than males) in comparison to other microtines or mammals in general (Bondrup-Nielsen & Ims 1990; Ralls 1976; Schulte-Hostedde 2007). At our field study site, male pups (mean ± SD, 1·81 ± 0·22 g) are on average 2% larger than female pups (1·77 ± 0·25 g, *n* = 103 litters; Koivula *et al.* 2003) and, as young adults (40 days), males (14·69 ± 2·18 g) are *c*. 5% larger than females (13·97 ± 1·95 g; Koivula *et al.* 2003). In the laboratory, SSD is reduced at birth (mean ± SD, males: 1·81 ± 0·22 g, females: 1·77 ± 0·25 g) and almost non-existent at weaning (day 20, males: 11·20 ± 1·31 g, females: 11·13 ± 1·41 g). However, laboratory raised males (19·22 ± 2·94 g) become 10% larger than females (17·32 ± 2·38 g) by the time they reach early adulthood. In this study, the SSD at birth was also very small (males: 1·84 ± 0·21 g, females: 1·82 ± 0·19 g).

The bank voles used in this experiment were mature individuals taken from third and fourth generations of a captive colony, originally stocked from our field study site located in Konnevesi, central Finland. The majority of females (80%) had given birth at least once before the study. The voles were housed individually in mouse cages (43 × 26 × 15 cm) and maintained on a 16L:8D photoperiod at 20 ± 2 °C. Wood shavings and hay were provided as bedding, while food (Labfor 36; Lactamin AB, Stockholm, Sweden) and water were provided *ad libitum.*

### Manipulation of rearing environment and sex ratio

The experiment started in early May (beginning of breeding season in nature) by mating females with randomly chosen males. After parturitions, newborn pups were immediately sexed, weighed to the nearest 0·01 g and individually marked. The mothers were weighed and their head widths were measured for the estimation of body condition as standardized residuals from the linear ordinary least-squares regression of body mass on body size (Schulte-Hostedde *et al.* 2005). Females were mated in postpartum oestrus with randomly chosen males (proven stud), because being pregnant while lactating is a natural state for females in the wild (Bronson 1989). Experimental litters were created by cross-fostering pups within 1 day of parturition and they consisted of the same aged pups, each originating from different mothers. Mothers (*n* = 72) were randomly assigned to two groups of litter sex ratio manipulation: (i) all-male and (ii) all-female litters. Moreover, rearing environment was manipulated by either (i) reducing or (ii) enlarging the original litter size by two pups (see Oksanen *et al.* 2003). The assignment of mothers to different manipulation groups was carried out so that their characteristics (body weight and condition, original litter size and sex ratio) did not differ significantly between the treatments (anova, all *P*> 0·2). Thus, the study design was a two-by-two factorial experiment, where both the sex ratio and growing environment of the offspring were manipulated. Using all-male and all-female litters was chosen as the most powerful design when testing for an effect, although the occurrence of single sex litters is relatively rare in natural populations (10% in Koivula *et al.* 2003). Bank vole females easily accept foreign pups, as it has been found that the growth does not differ between the pups that are cared by natural vs. foster mothers (e.g. Mappes, Koskela & Ylönen 1995a; Koskela *et al.* 1998). Throughout the text, ‘mother’ refers to foster mother (suckling mother) of the offspring. The sexing of newborn pups (by the help of visual cues and the length of anogenital distance) was proven very accurate at the weaning age; only 4 males out of 367 pups used in the study were incorrectly sexed as females.

### Offspring defence

The intensity of offspring defence was assessed 4 days after manipulation of litter sex and size using a protocol similar to the one described in Koskela *et al.* 2000. Whole litter was taken from the nest, placed together with its bedding into a small cage (15 × 10 × 7 cm) and positioned at the centre of the arena (0·6 m^2^). The (foster) mother of the pups was then released into the arena, and after a 3-min familiarization period beginning from when it first noticed the pups, a strange male was also introduced into the arena. The behaviour of the female–male pair was recorded on a videotape for 10 min from the time the female first noticed the male. The recordings were analysed by two people unaware of the female assignments to the experimental treatments. We measured the female’s aggressive behaviour towards the male by counting (i) number of attacks/threats (vole leaps at its opponent, often followed by chasing/defensive postures, such as upright stance often followed by lunges and vocalization); (ii) number of chases (running after opponent, usually after an attack); (iii) number of fights (physical wrestling, vole pair rolling around the arena); (iv) total combined time used for the three behaviours and (v) duration of male activity towards the female (i.e. the time the male approached the female or the cage with pups). The intensity of offspring defence was determined for 45 mothers.

### Milk production

Milk production of females was assessed 5 days after manipulation of litter sex and size using the method described in Oksanen *et al.* (1999). The pups were separated from their mother for 3 h to let them consume the milk in their stomachs. Then the mother was allowed to nurse the pups for 2 h. The pups were weighed before and after the nursing to determine the amount of milk they had received. We used two measures of milk production: total amount of milk produced (sum of mass increases in a litter) and milk produced per pup (average mass increase of a pup in a litter).

### Subsequent breeding success of females

To study the fecundity cost of rearing manipulated litters for the females, the proportion of females producing a second litter, duration of their subsequent pregnancy and characteristics of the second litters were measured.

### Data analyses

Generalized linear models and generalized linear mixed models (GLMM) were used to analyse the data. GLMM allows for offspring to be used as nested data points by controlling for foster mother identity as a random blocking factor in the model (Paterson & Lello 2003). Offspring growth during nursing (measured at birth and 20 days of age) was studied using proc mixed, sas® where the weight of the individual offspring at 20 days of age was used as a dependent variable, while the litter sex (fixed), offspring number treatment (fixed), their interaction and the mother identifier (random) were added as independent variables. Weight at birth and condition of mother were entered in the model as covariates. Milk intake of individual pups was analysed similarly as offspring growth but only the condition of mother was used as a covariate.

The counts of the attacks/threats, chases and fights were ln(*x* + 1) transformed to normalize their distribution and a principal component analysis was run to summarize these variables. The first component extracted from the data set accounted for 70·2% of the total variance. The three variables had positive loadings: attacks/threats 0·855, chases 0·743, fights 0·908, and the component was described as ‘aggressive behaviour’. Total defence time and the duration of male activity had positively skewed distributions, and thus the variables were sqr(*x* + 0·5) transformed before the analyses. Those variables were analysed using anova with the two fixed treatments and their interaction. The duration of male activity towards the female did not depend upon manipulation group (anova, litter sex: *F*_1,41_ = 0·21, *P* = 0·647; litter size: *F*_1,41_ = 0·07, *P* = 0·80; interaction: *F*_1,41_ = 0·10, *P* = 0·749). Thus, male behaviour was not included in the models as a separate factor. In this study, we were primarily interested in the interaction between litter size and sex ratio treatments, so this interaction was retained in the models even if not significant. The analyses were performed using sas® v. 9.1 (SAS Institute Inc, Cary, NC) and spss 12.0.1 (SPSS Inc, Chicago, Illinois) software.

## Results

### Offspring growth

**Fig. 1:**
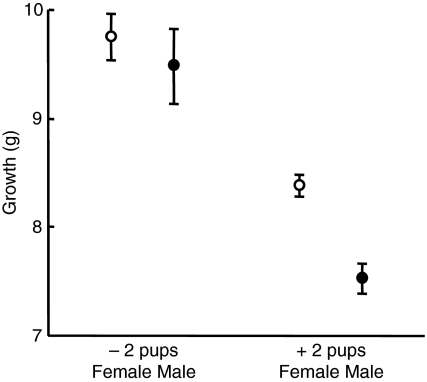
Offspring growth [weight change (g) from birth until weaning, mean ± SE] was significantly faster in reduced litters than enlarged litters and in daughters than sons.

Both litter sex ratio and offspring number manipulations significantly affected the growth of the pups during nursing. Female offspring grew faster than males, while offspring in reduced litters (that represent a good environment) showed faster growth than offspring in enlarged litters (a poor rearing environment) (Table 1; Fig. 1). Maternal condition did not have a significant effect or interactions with treatments on offspring growth (condition × litter sex: *F*_1,40·2_ = 2·42, *P* = 0·128; condition × litter size: *F*_1,43·3_ = 1·40, *P* = 0·242; three-way interaction: *F*_1,42·1_ = 0·14, *P* = 0·709) and was therefore removed from the final model.

**Table 1:** Generalized linear mixed model on size of offspring at weaning age in different treatment groups

	Estimate ± SE	d.f.	*F*	*P*
Intercept	0·772 ± 0·037			
Litter size	0·082 ± 0·018	1, 45·9	27·72	<0·0001
Litter sex	0·042 ± 0·017	1, 45·9	4·03	0·050^a^
Litter size × litter sex	−0·034 ± 0·025	1, 45·8	1·85	0·180
Weight at birth	0·523 ± 0·077	1, 217	46·07	<0·0001

Mother identity was included in the model as a random factor (estimate ± SE: 1·624 × 10^−3^ ± 0·407 × 10^−3^, *Z* = 3·99, *P* < 0·0001), weight at birth as a covariate. Satterthwaite approximation for the denominator degrees of freedom used.

a If the non-significant interaction term is removed from the model, Litter sex manipulation *P* = 0·039.

### Milk production

**Fig. 2:**
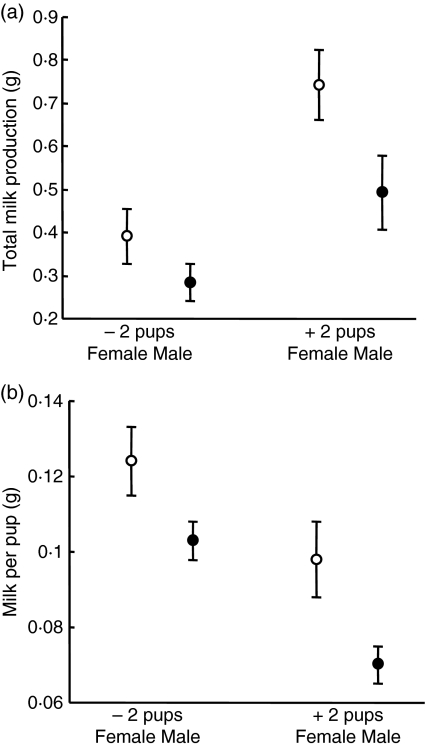
(a) Mothers produced significantly more milk (in grams, mean ± SE) for female litters and for enlarged litters. (b) An average female pup received more milk (in grams, mean ± SE) than a male pup and in enlarged litters, a pup received less milk than in reduced litters.

Total milk produced by the mother was significantly affected by maternal condition as well as manipulations of litter sex ratio and offspring number (Table 2). Mothers produced significantly more milk for female litters and for enlarged litters; however, these effects did not interact with maternal condition (condition × litter sex: *F*_1,65_ = 0·601, *P* = 0·441; condition × litter size: *F*_1,65_ = 3·09, *P* = 0·084; three-way interaction: *F*_1,64_ = 2·83, *P* = 0·100; Fig. 2a). Average female pup received more milk than a male pup, but pups in enlarged litters got less milk than in reduced litters (Table 2; Fig. 2b). Again, maternal condition did not interact significantly with the treatments (condition × litter sex: *F*_1,56·2_ = 1·19, *P* = 0·280; condition × litter size: *F*_1,60_ = 0·65, *P*= 0·425; three-way interaction: *F*_1,58·6_ = 2·80, *P* = 0·100). The amount of milk produced predicted the size of offspring at weaning (partial correlation, controlling for litter size: *r*_p_ = 0·33, *n* = 48, *P* = 0·018).

**Table 2:** Two-way anova and generalized linear mixed model for milk production of mothers in different treatment groups

	Estimate ± SE	d.f.	*F*	*P*
Total milk production				
Intercept	0·978 ± 0·033			
Litter size	−0·091 ± 0·046	1, 67	13·515	<0·001
Litter sex	0·114 ± 0·045	1, 67	6·307	0·014
Litter size × litter sex	−0·060 ± 0·064	1, 67	0·875	0·353
Mother condition	0·052 ± 0·016	1, 67	9·124	0·004
Milk per pup^a^				
Intercept	0·753 ± 0·008			
Litter size	0·020 ± 0·011	1, 62·7	4·34	0·041
Litter sex	0·021 ± 0·010	1, 63·3	4·95	0·030
Litter size × litter sex	−0·008 ± 0·016	1, 63·1	0·24	0·627
Mother condition	0·006 ± 0·004	1, 59·1	2·11	0·151

Satterthwaite approximation for the denominator degrees of freedom used.

a Mother identity was included in the model as a random factor (estimate ± SE: 0·85 × 10^−3^ ± 0·19 × 10^−3^, *Z* = 4·52, *P* < 0·0001).

### Offspring defence

**Fig. 3:**
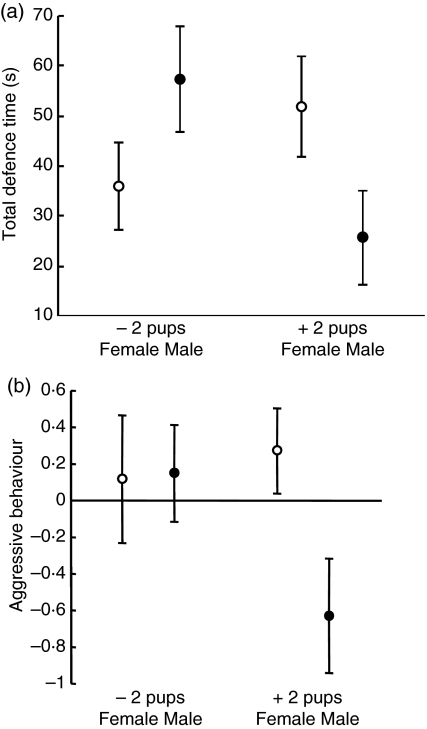
(a) Total defence time (in seconds, mean ± SE) of the mothers did not differ significantly between male and female offspring in reduced litters whereas mothers defended daughters more actively than sons in enlarged litters. (b) Aggressive behaviour of mothers (PCA component, mean ± SE) did not reach a statistically significant difference between the treatments.

There was a significant interaction between the litter sex and offspring number manipulations for the duration of female defence activity (Table 3; Fig. 3a). According to (Bonferroni adjusted) univariate tests, there was no significant difference in the total defence time of the mothers between male and female litters in a reduced litter treatment (*F*_1,41_ = 1·91, *P* = 0·175) whereas mothers defended daughters longer than sons in enlarged litter treatments (*F*_1,41_ = 5·12, *P* = 0·029). Maternal condition did not have a significant main effect or interaction with female defence activity in different treatments (condition × litter sex: *F*_1,38_ = 0·56, *P* = 0·460; condition × litter size: *F*_1,38_ = 1·31, *P* = 0·260; three-way interaction: *F*_1,37_ = 0·01, *P* = 0·921).

**Table 3:** Two-way anova of offspring defence of mothers in different treatment groups

	Estimate ± SE	d.f.	*F*	*P*
Total defence time				
Intercept	4·317 ± 0·852			
Litter size	2·710 ± 1·154	1, 41	0·61	0·441
Litter sex	2·612 ± 1·154	1, 41	0·43	0·515
Litter size × litter sex	−4·166 ± 1·612	1, 41	6·68	0·013
Aggressive behaviour				
Intercept	−0·630 ± 0·307			
Litter size	0·780 ± 0·416	1, 41	1·15	0·289
Litter sex	0·900 ± 0·416	1, 41	2·22	0·144
Litter size × litter sex	−0·935 ± 0·581	1, 41	2·59	0·115

Analysis of the data on aggressive behaviour (PCA component) (Fig. 3b) fails to support the arguments presented above, as the interaction between the treatments does not reach statistical significance (Table 3). Again, maternal condition did not have significant effects (condition × litter sex: *F*_1,38_ = 0·45, *P* = 0·507; condition × litter size: *F*_1,38_ = 0·81, *P* = 0·373; three-way interaction: *F*_1,37_ = 0·07, *P* = 0·798).

### Subsequent breeding success of females

The probability of subsequent breeding was over 80 percent in all of the treatment groups and did not depend upon experimental treatments (proc glimmix, litter size: *F*_1,49_ = 0·69, *P* = 0·411; litter sex: *F*_1,49_ = 0·54, *P* = 0·468; interaction non-significant). Likewise, maternal condition did not affect the probability of breeding (main effect of condition: *F*_1,47_ = 2·49, *P* = 0·122; interactions: *P* > 0·8).

Females rearing-enlarged litters had a significantly prolonged subsequent pregnancy in comparison to females nursing-reduced litters (22·2 vs. 19·8 days respectively), whereas litter sex ratio did not affect the delay of parturition (anova, litter size: *F*_1,33_ = 58·93, *P* < 0·001; litter sex: *F*_1,33_ = 2·00, *P* = 0·166; interaction: *F*_1,33_ = 0·10, *P* = 0·751). Maternal condition did not affect the delay of second parturition (condition × litter sex: *F*_1,30_ = 0·04, *P* = 0·835; condition × litter size: *F*_1,30_ = 0·14, *P* = 0·715; three-way interaction: *F*_1,29_ = 0·87, *P* = 0·359).

The subsequent litter size was significantly larger for females in better condition but was not affected by the treatments (ancova, litter size: *F*_1,38_ = 0·05, *P* = 0·824; litter sex: *F*_1,38_ = 2·70, *P* = 0·108; interaction: *F*_1,38_ = 0·041, *P* = 0·906; condition: *F*_1,38_ = 7·14, *P* = 0·011). Moreover, the interactions between maternal condition and treatments were not statistically significant (condition × litter sex: *F*_1,36_ = 0·56, *P* = 0·461; condition × litter size: *F*_1,36_ = 0·01, *P* = 0·916; three-way interaction: *F*_1,35_ = 0·75, *P* = 0·394).

The sex ratios of subsequent litters did not differ significantly between the treatments (events-trials, proc genmod, litter size: *F*_1,37_ = 0·05, *P* = 0·820; litter sex: *F*_1,37_ = 0·28, *P* = 0·603; interaction: *F*_1,37_ = 1·33, *P* = 0·257).

## Discussion

We used a novel experimental design to study the optimal maternal investment between different offspring sexes in a polygynandrous small mammal. Bank voles actively responded to a manipulation of the offspring sex ratio by differentiating maternal care between sons and daughters. The several measures of maternal investment (milk production, pup defence intensity and offspring growth) revealed that mothers clearly allocated more resources to female rather than male offspring independent of their own condition. This is in agreement with the predictions based on the bank vole life history: there will be selection for greater investment in daughters rather than sons, as larger size seems to be more important for female reproductive success in this species.

Manipulation based on reduction or enlargement of the original litter size immediately after birth has been a classic experimental design when studying the costs of reproduction and other reproductive trade-offs (e.g. Mappes *et al.* 1995a; Humphries & Boutin 2000; Oksanen *et al.* 2003). This method is not without complications (e.g. McGuire & Bemis 2007), but can serve as a powerful tool to investigate the mother’s allocation decisions between offspring and their own body condition. In the present experiment, enlargement of litter size successfully manipulated the mothers to increase their milk production (and caused the delay in their second parturition), but this increase was not sufficient to meet the demands of offspring. Current theory about the limits to maximum sustainable level of energy intake and further reproductive effort suggests that the limits are imposed by the capacity of the animal to dissipate body heat generated as a by-product of processing food and producing milk (Król, Murphy & Speakman 2007). As our study was carried out under a constant temperature, it may mask effects that would become visible in a more natural environment. Another potential confounding factor is a lack of mixed-sex litters from the experimental design. Single sex litters are relatively rare in nature (see Material and methods), and it is unknown how mothers would allocate their care between sons and daughters in mixed-sex litters.

Studies on sex-biased investment in small mammals are surprisingly scarce and have typically concentrated on the adjustment of sex ratios, not on energy allocation between the sexes (McShea & Madison 1986; Lambin 1994; Aars, Andreassen & Ims 1995; Bond *et al.* 2003; review in Sikes 2007; but see McClure 1981; Clark, Waddingham & Galef 1991; Koskela *et al.* 2004). Those studies typically observed female-biased maternal investment, as in the present study, and suggested that sex ratio variation in relation to reproductive competition among females is widespread among small rodents with female-biased maternal investment when opportunities for rapid maturation of females are the greatest. Consequently, even though the present study concerned maternal allocation during nursing and not sex ratios, it is supported by earlier findings that microtine females possess mechanisms that enable them to adjust their investment between sons and daughters adaptively (e.g. Clark *et al.* 1991; Koskela *et al.* 2004; review in McGuire & Bemis 2007). An important characteristic of maturing individuals is their competitive ability, which is often suggested to relate to size in mammals. As described in the Introduction section, Bondrup-Nielsen & Ims (1990) proposed that in microtine rodents, the sex competing for a resource will be under selection for larger size. In several vole species, male reproductive success is largely determined by active searching and direct competition for sexually receptive females (e.g. Kawata 1985; Ostfeld 1985). However, selection for higher mobility in males should in fact select for smaller size (because of energetic reasons), whereas intraspecific competition in territorial females should select for larger size (Bondrup-Nielsen & Ims 1990). Obviously, higher mobility could also select for non-size-related factors, such as a higher metabolism or testosterone level in males. In fact, selection for body mass in male bank voles has not been observed, as testosterone level rather than body size determines their reproductive success (Mills *et al.* 2009; but for survival, see Yoccoz & Mesnager 1998). Thus, when considering the evolution of SSD in the bank vole, selection should favour larger females rather than larger males, which would then show up in sex-specific maternal investment. And although this idea is not supported by our field data on relative sizes of males and females (see Material and methods), we suggest, in accordance with Bondrup-Nielsen & Ims (1990), that this pattern could be a general rule in highly polygynous, but weakly dimorphic small mammals where females are territorial.

In their extensive review, Cockburn *et al.* (2002) listed 11 adaptive hypotheses that have been applied to explain sex ratios in birds and mammals. The most influential of these, the Trivers & Willard (1973) hypothesis, predicts that parents in good condition should bias their allocation towards the sex that derives the greater reproductive payoff from a given level of investment. Does the bank vole system fill the underlying assumptions [described carefully in Hewison & Gaillard (1999) and Cameron & Linklater (2002)] of the T–W model? In the present study, maternal condition predicted the quality of maternal care, that is, the amount of milk produced, which further correlated positively with the size of the offspring at independence (see also Oksanen *et al.* 1999). Combined with an earlier finding that the differences in the size of bank vole young are often found to persist into adulthood (e.g. Koskela 1998), the first two assumptions of T–W hypothesis seem to hold. However, as pointed out by Hewison & Gaillard (1999) and Cockburn *et al.* (2002), demonstrating unequivocally the third assumption, that a given unit of investment has a different impact on the reproductive success of sons relative to daughters, is often difficult to prove. In bank vole, the variation in body size has differential effects on males and females (see Introduction), consistent with the conditions of the third assumption. Now given that the assumptions of the model are met, our results do not provide support for the narrow sense T–W hypothesis (Cockburn *et al.* 2002), which states that maternal investment is biased towards the sex with higher variation in reproductive success. In contrast, we show that the mothers invested more in daughters.

Leimar’s (1996) modification of the T–W hypothesis, rather than focusing on offspring reproductive success, shifted interest to the concept of reproductive value, defined as the proportion of the expected contribution of an individual in a current circumstance to the future gene pool of the population. Using state-dependent life-history theory, he showed that high-quality mothers sometimes should prefer daughters even if sons would have a higher reproductive success; this will happen when offspring quality is strongly influenced by the mother’s but not father’s quality. Even when the present study cannot exceed its understandable limitations, it is possible to discuss the results from this perspective using previous knowledge of the study system. As typical for small mammals, the size of bank vole offspring at independence is strongly determined by the amount of maternal care during nursing (current study; Mappes *et al.* 1995a; Koskela *et al.* 1998; Oksanen *et al.* 2003) and size often affects future fitness, especially in daughters (see Introduction). Moreover, the father’s quality (measured as mating success and body size) has not been found to have any positive impact on offspring number or size (at birth or at independence) and bank vole mothers do not differentiate maternal care according to a mate’s quality (Oksanen *et al.* 1999). However, as male mating success is known to be heritable in the bank vole (Oksanen *et al.* 1999), it has obvious effects on the reproductive value of sons, which are then apparently not possible to improve by increasing maternal effort. Thus, in line with Leimar’s (1996) predictions, our study contributes to the evidence that maternal qualitative investment can be more crucial for daughters than sons, as larger size at independence is more important for the reproductive value of female offspring. Moreover, bank vole mothers should benefit from biasing offspring sex ratio towards sons if mated with good males, a hypothesis that remains to be addressed in future studies.

In light of our results, the prediction that male offspring receive more investment from their high-quality mothers in polygynous mammals might be more species-specific than previously considered. Our study reaffirms the need to investigate sex-biased investment with an attention to the life history of each study species, and recognizes the fact that, in any system, multiple hypotheses may be at work.
